# Increased interleukin-6 levels associated with malaria infection and disease severity: a systematic review and meta-analysis

**DOI:** 10.1038/s41598-022-09848-9

**Published:** 2022-04-08

**Authors:** Polrat Wilairatana, Wanida Mala, Giovanni De Jesus Milanez, Frederick Ramirez Masangkay, Kwuntida Uthaisar Kotepui, Manas Kotepui

**Affiliations:** 1 Department of Clinical Tropical Medicine, Faculty of Tropical Medicine, Mahidol University, Bangkok, Thailand; 2 Medical Technology, School of Allied Health Sciences, Walailak University, Tha Sala, Nakhon Si Thammarat, Thailand; 3 Department of Medical Technology, Faculty of Pharmacy, University of Santo Tomas, Manila, Philippines

**Keywords:** Infectious-disease diagnostics, Parasitology

## Abstract

Interleukin-6 (IL-6) is generated by immune cells during infection with malaria parasites and they are associated with the immunopathogenesis of malaria. The present systematic review and meta-analysis aimed to compare the differences in IL-6 levels between several groups of patients with malaria and healthy control groups. The systematic review was registered at PROSPERO with a registration number: CRD42021290753. Systematic literature searches were conducted in PubMed, Web of Science, and Scopus until November 7, 2021 to obtain studies that documented IL-6 levels in patients with malaria. The quality of the included studies was assessed using critical appraisal tools from the Joanna Briggs Institute. Differences in the mean IL-6 levels among patients with: (1) severe and non-severe malaria, (2) uncomplicated malaria and controls, (3) uncomplicated and asymptomatic malaria, (4) asymptomatic malaria and healthy controls, and (5) those that died or survived were estimated using a random-effects model. Forty-three of 1,969 studies were included in the systematic review. Results of the meta-analysis showed that patients with severe malaria had higher mean IL-6 levels than those with non-severe malaria [*P* = 0.04, weight mean difference (WMD) = 96.63 pg/mL, 95% confidence interval (CI) = 0.88 − 19.38 pg/mL, *I*^2^ = 99.9%, 13 studies]. Patients with uncomplicated malaria had higher mean IL-6 levels than the controls (*P* < 0.001, WMD = 42.86 pg/mL, 95% CI = 30.17 − 55.56 pg/mL, *I*^2^ = 100%, 17 studies). No differences in the mean levels of IL-6 were found between patients with uncomplicated malaria and those with asymptomatic malaria (*P* = 0.063, WMD = 42.07 pg/mL, 95% CI =  − 2.23 pg/mL to − 86.37 pg/mL, *I*^2^ = 99.1%, 8 studies), or between patients with asymptomatic malaria and healthy controls (*P* = 0.45, WMD = 1.67 pg/mL, 95% CI =  − 2.73 pg/mL to − 6.07 pg/mL, *I*^2^ = 98.1%, 2 studies). A higher mean level of IL-6 was observed in patients who died compared with the levels of those who survived (*P* = 0.007, WMD = 1,399.19 pg/mL, 95% CI = 384.16 − 2,414.2 pg/mL, *I*^2^ = 93.1%, 4 studies). Our meta-analysis of the pooled evidence can be used to guide future studies in which IL-6 levels are measured during malaria outbreaks to monitor malaria severity. Heterogeneity of the effect estimate among the included studies was the main limitation of this analysis. In conclusion, significantly increased levels of IL-6 were observed in patients with severe malaria compared with those in patients with non-severe malaria, which indicates that IL-6 is a candidate marker for severe malaria. Future studies should investigate the sensitivity and specificity of increased IL-6 levels to determine the effectiveness of assessments of IL-6 levels monitoring of malaria infection and severity.

## Introduction

Cells that provide innate and adaptive immunity in cellular and antibody-mediated immunity play critical roles in immune responses to malaria parasites and this mediates the immunopathogenesis of malaria^1^. Immunity to malaria parasites is affected by the patient’s age and degree of exposure to malaria, and people living in malaria-endemic areas develop immunity against symptomatic malaria and this leads to asymptomatic infection^1^. Proinflammatory cytokines exacerbate the disease of malaria whereas anti-inflammatory cytokines reduce inflammation and promote healing^2^. Proinflammatory cytokines are involved in the clearance of *Plasmodium falciparum* and high levels *of P. falciparum* have been documented in the pathogenesis of severe malaria^3–6^. Increased levels of proinflammatory cytokines such as interleukin-1 beta (IL-1β), IL-6, IL-8, IL-10, IL-12, IL-13, IL-31, IL-33, and tumor necrosis factor alpha (TNF-α) have been reported to be related to clinical malaria or severe malaria^7–9^.

IL-6 is generated at sites of infection and inflammation by immune cells^10^, adipocytes^11^, and endothelial cells^12^. IL-6 induces acute phase proteins such as fibrinogen, C-reactive protein, haptoglobin serum amyloid A, and α1-antichymotrypsin^13^. Furthermore, IL-6 promotes the differentiation of naïve CD4^+^ T cells, which suggests it has an essential role in the development of adaptive immunity^10^. It has been reported that, during immunopathogenesis of malaria, IL-6 is regulated by TNF-α and co-operates with other inflammatory mediators to control parasitemia^14–16^. Although IL-6 had been reported to have different roles in the pathogenesis of malaria infection and varies with disease severity^4,7,16–18^, these studies enrolled a limited number of participants with severe malaria and therefore the conclusions are questioned. The present systematic review and meta-analysis compared the differences in IL-6 levels among several groups of patients with malaria and control groups. Our meta-analysis of the pooled evidence might suggest better analysis of measurements of IL-6 during malaria infections in future studies to monitor malaria severity.

## Methods

This systematic review was reported following the Preferred Reporting Items for Systematic Reviews and Meta-analyses (PRISMA) guidelines^19^. The systematic review was registered at PROSPERO and the registration number is CRD42021290753.

### Database searches

Systematic literature searches were conducted in PubMed, Web of Science, and Scopus with the last date of search on November 7, 2021 to obtain studies that documented IL-6 levels in patients with malaria. The search was restricted to the English language and the following search terms were used: “(malaria OR plasmodium) AND (IL-6 OR IL6 OR “Interleukin 6” OR “B-Cell Stimulatory Factor 2” OR “Differentiation Factor-2” OR BSF-2 OR “IFN-beta 2” OR “Interferon beta-2” OR MGI-2). A full description of the search strategy is shown in Table S1.

### Definitions

Severe falciparum malaria is due to the presence of *P. falciparum* asexual parasitaemia with one or more of the following complications: impaired consciousness, multiple convulsions, acidosis, hypoglycemia, severe malarial anemia, renal impairment, jaundice, pulmonary edema, significant bleeding, shock, and hyperparasitaemia^20^. Uncomplicated malaria includes patients who present with symptoms of malaria and have evidence of *P. falciparum* asexual parasitaemia but without the complications listed by the WHO. Controls were divided into febrile and healthy control participants. Febrile participants who were enrolled in the study were participants who were febrile but without malaria parasitaemia in the blood; healthy controls were participants who lived in the same area but did not have diseases.

### Eligibility criteria

PICo (P: participants, I: outcome of interest, C: controls, O: difference in outcome of interest) was applied to identify eligible studies. 1) P: patients with malaria either with or without signs or symptoms of malaria, but with malaria parasites, which were detected by microscopy, rapid diagnostic tests, or molecular methods, or a combination of these methods; 2) I: IL-6 levels measured with an enzyme-linked immunosorbent assay (ELISA) or bead-based assay; 3) C: febrile or healthy participants living in the same area who tested negative for malarial parasites; and 4) O: differences in IL-6 levels between malaria patients and controls. The following studies were excluded for the following reasons: use of an animal model, in vitro studies, qualitative data on IL-6, genetic studies (gene expression analysis), reviews or systematic reviews, pregnancy or cord blood analysis, full text unavailable, IL-6 measurement after patients were treated, and non-English articles.

### Study selection

Potentially relevant studies were retrieved from databases through searches and selected studies were stored in EndNote X9 (Clarivate Analytics, Philadelphia, PA, USA) for analysis. The process of study selection was as follows: (1) duplicate studies were excluded; (2) titles and abstracts of the remaining studies were screened; (3) non-relevant titles and abstracts were excluded; (4) the remaining studies were examined to identify the full-text; (5) studies with full text were selected based on eligibility criteria; and (6) studies that did not meet the criteria for a specific reason were excluded. Two authors (MK and WM) independently selected studies and any disagreement between the two authors was resolved by consultation with a third author (PW).

### Data extraction

After eligible studies were selected based on specific criteria, the following data were extracted using a standardized Excel spreadsheet: author/s, year of publication, study design, study location and year, characteristics of participants and participant numbers, *Plasmodium* spp., age of participants, male percentage, IL-6 levels (pg/mL), parasite count, method of malaria detection, and method of IL-6 measurement. Two authors (MK and WM) independently extracted studies and any disagreement between the two authors was resolved by discussion for consensus.

### Quality assessment

Two authors (MK and WM) assessed the quality of the included studies independently using critical appraisal tools of the Joanna Briggs Institute (JBI)^21^. These tools include a different number of items based on different study designs. The JBI tool for a critical appraisal checklist for cohort studies has 11 items (groups of populations, exposures measured, valid and reliable way of exposure, confounding factors, strategies to control for confounding factors, free of the outcome at the start of the study, outcomes measured, follow-up time, completion of follow-up, strategies to address incomplete follow-up, appropriateness of statistical analysis). The JBI tool for critical appraisal checklist for case–control studies has 10 items (i.e., groups of populations, matching of cases and controls, criteria used for the identification of cases and controls, valid and reliable method of exposure, same method for exposure used for cases and controls, confounding factors, strategies to deal with confounding factors, measurement of outcomes, exposure period of interest, and appropriateness of statistical analysis). The JBI tool for critical appraisal checklist for randomized controlled trials (RCTs) had 13 items (true randomization, allocation to treatment groups, similarity of treatment groups at baseline, participants blind to treatment assignment, delivering treatment blind to treatment assignment, outcome assessors blind to treatment assignment, treatment groups treated identically except for the intervention of interest, completeness of follow-up, participants analyzed in the groups to which they were randomized, the same outcomes measured for treatment groups, reliable measurement of outcomes, appropriateness of statistical analysis, appropriateness of trial design). The quality of each study was rated as low (< 50 percentile), moderate (50–75 percentile), or high (> 75 percentile) according to a summary score, which was divided by a total score and then multiplied by 100 to obtain a percentile (Table S2).

### Data analysis

Differences in the mean IL-6 levels between patients with: (1) severe and non-severe malaria, (2) uncomplicated malaria and controls, (3) uncomplicated and asymptomatic malaria, (4) asymptomatic malaria and healthy controls, and (5) those who died or survived were estimated using a random-effects model and the DerSimonian-Laird method^22^. The results are shown as the weight mean difference (WMD) based on the inverse variance approach for measuring weight and the 95% confidence interval (CI). Heterogeneity among the included studies was assessed with *I*^2^ statistics for inconsistency with *I*^2^ values > 25%, 25–75%, and < 75% indicating as low, moderate, and high heterogeneity, respectively^23^. Publication bias was assessed by visual inspection of a funnel plot and with Egger’s test. A contour-enhanced funnel plot was also constructed to determine whether the asymmetry of the funnel plot was due to publication bias or other factors. If the asymmetry was due to publication bias, the trim and fill method^24^ was applied to correct the estimate of the effect. Subgroup analysis was based on the characteristics of the included studies (study location, method of malaria detection, method of IL-6 measurement) and the characteristics of participants (*Plasmodium* spp., age group). Univariate meta-regression analyses were performed including the characteristics of the studies and characteristics of participants to identify the source(s) of heterogeneity among the included studies. All statistical analyses were carried out with Stata version 14 (StataCorp LLC, Texas, USA).

## Results

### Search results

**Figure 1:**
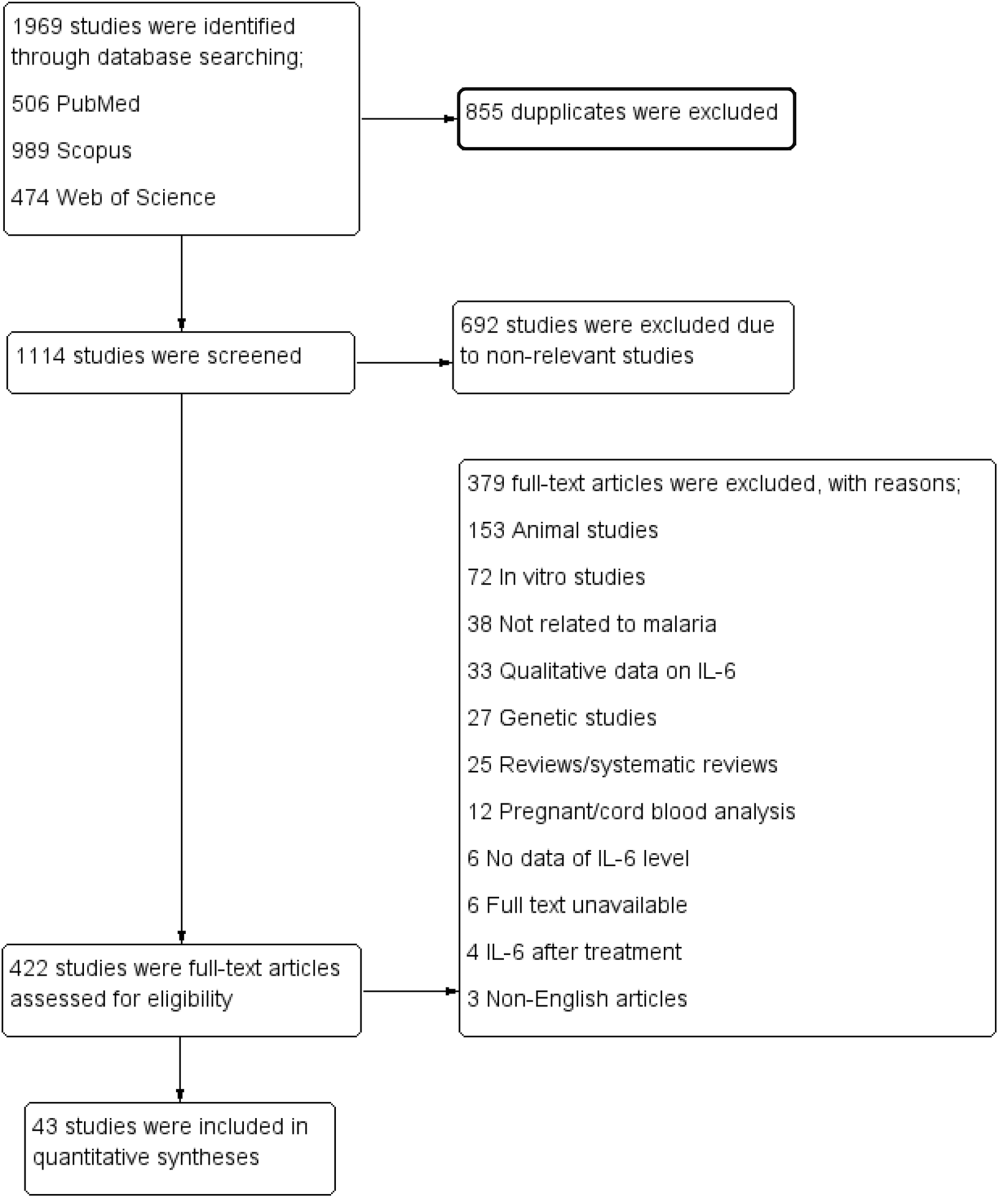
Study flow diagram.

Overall, 1,969 studies were identified from PubMed (506 studies), Web of Science (474 studies), and Scopus (989 studies). After 855 duplicates were removed, the title and abstracts of 1,114 studies were screened. A total of 692 studies that were non-relevant were excluded, leaving 422 studies for full-text examination. Examination of 422 studies resulted in the exclusion of 379 full-text articles based on the following criteria: 153 animal studies; 72 in vitro studies; 38 not related to malaria; 33 with only qualitative data on IL-6; 27 genetic studies; 25 reviews/systematic reviews; 12 pregnant/cord blood analysis; six with no data on IL-6 levels; six with full text unavailable; four with IL-6 measured after treatment; and three non-English articles (Fig. 1). Studies that included pregnant/cord blood analysis were excluded from the review because the participants were not of interest and these participants may have baseline cytokine levels that differed from other groups, which might have increased the heterogeneity of the estimate of the effect among the included studies.

### Characteristics of the included studies

The characteristics of the included studies are summarized in Table 1. Most of the included studies were published between 2011 and 2021 (26, 60.5%) and fewer studies were published between 2001 and 2010 (9, 20.9%) and 1991–2000 (8, 18.6%). Most of the included studies were conducted in Africa (27, 62.8%) followed by Asia (9, 20.9%), America (5, 11.6%), and Europe (2, 4.65%). Most of the included studies were prospective observational studies (19, 44.2%), and the remaining study types included cross-sectional studies (16, 37.2%), case-controlled studies (6, 14%), and randomized controlled trials (RCTs) (2, 4.6%). The studies enrolled patients with *P. falciparum* (32, 74.4%), *P. vivax* (4, 9.3%), *P. falciparum*/*P. vivax* (3, 6.98%), *P. falciparum*/*P. knowlesi* (1, 2.3%), *P. falciparum*/*P. vivax*/*P. ovale* (1, 2.3%), *P. falciparum*/*P. vivax*/mixed infection (1, 2.3%), and *P. falciparum*/*P. vivax*/*P. ovale*/*P. malariae* (1, 2.3%). Some studies enrolled children (20, 46.5%), adults (16, 37.2%), all age groups (6, 14%), and not specified (1, 2.3%). Most of the studies (31, 72.1%) used only microscopy for malaria detection although microscopy/PCR (5, 11.6%), microscopy/rapid diagnostic test (RDT; 5, 11.6%), and microscopy/RDT/PCR (2, 4.65%) were also used. An ELISA was the most common method used for IL-6 measurements (30, 69.8%), followed by a bead-based assay (13, 30.2%). Details of the studies are shown in Table S2.

**Table 1:** Characteristics of the included studies.

Parameters	Number of study (%)
**Publication years**	
1991–2000	8 (18.6%)
2001–2010	9 (20.9%)
2011–2021	26 (60.5%)
**Study locations**	
Africa	27 (62.8%)
Asia	9 (20.9%)
America	5 (11.6%)
Europe	2 (4.65%)
**Study designs**	
Prospective observational studies	19 (44.2%)
Cross-sectional studies	16 (37.2%)
Case control studies	6 (14%)
Randomized control trials	2 (4.6%)
***Plasmodium* species**	
*P. falciparum*	32 (74.4%)
*P. vivax*	4 (9.3%)
*P. falciparum****/****P. vivax*	3 (6.98%)
*P. falciparum*/*P. knowlesi*	1 (2.3%)
*P. falciparum/P. vivax/P. ovale*	1 (2.3%)
*P. falciparum/P. vivax/*Mixed infection	1 (2.3%)
*P. falciparum/P. vivax/P. ovale/P. malariae*	1 (2.3%)
**Age groups**	
Children	20 (46.5%)
Adults	16 (37.2%)
All age groups	6 (14%)
Not specified	1 (2.3%)
**Malaria detection**	
Microscopy	31 (72.1%)
Microscopy/PCR	5 (11.6%)
Microscopy/RDT	5 (11.6%)
Microscopy/RDT/PCR	2 (4.65%)
**IL-6 measurement**	
ELISA	30 (69.8%)
Bead-based assay	13 (30.2%)

*IL-6* interleukin-6, *ELISA* enzyme-linked immunosorbent assay.

### Quality of the included studies

Thirty-two studies (74.4%)^4,6,7,16–18,25–50^ were high quality, whereas 11 studies (25.6%)^51–61^ were moderate qualities (Table S3). As no low-quality study was found, all studies were included in the review.

### Differences in IL-6 levels between severe and non-severe malaria cases

**Figure 2:**
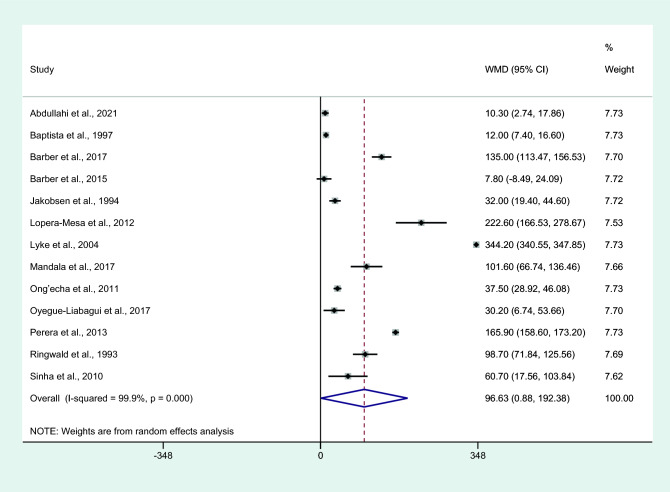
Differences in IL-6 levels between patients with severe malaria and non-severe malaria. Abbreviation: WMD, weighted mean difference; CI, confidence interval; black diamond symbol, point estimate; solid line in the middle of the graph at 0, zero effect size; Dashed red line: pooled WMD between the two groups; *I*^2^, level of heterogeneity; *P* = 0.00 or less than 0.05, significant heterogeneity.

Differences in IL-6 levels between patients with severe and non-severe malaria cases were estimated using the available data of 13 studies^4,6,7,16–18,25,28,29,34,41,45,61^. The results showed that patients with severe malaria had higher mean IL-6 levels than those with non-severe malaria (*P* = 0.04, WMD = 96.63 pg/mL, 95% CI = 4.322 − 188.89 pg/mL, *I*^2^ = 99.9%, 13 studies; Fig. 2). Meta-regression analyses of continents, malarial complications, parasitemia, age, male percentage, malaria diagnostic methods, and IL-6 measurement methods showed that these co-variates did not confound the effect estimate (*P* > 0.05).

**Figure 3:**
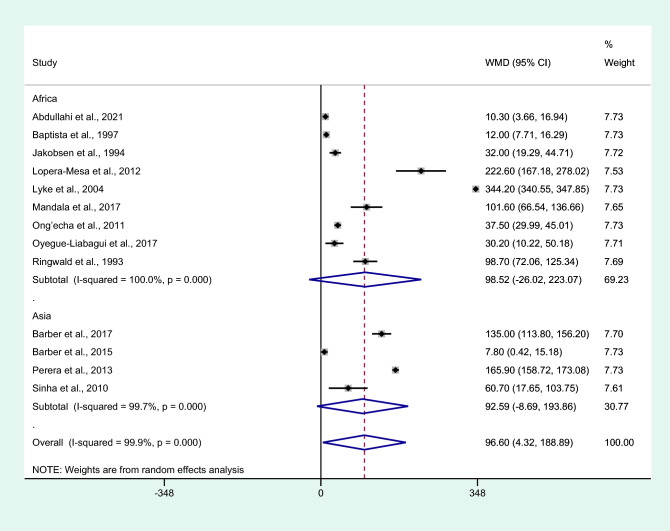
Differences in IL-6 levels between patients with severe malaria and non-severe malaria by continents. Abbreviation: WMD, weighted mean difference; CI, confidence interval; black diamond symbol, point estimate; solid line in the middle of the graph at 0, zero effect size; Dashed red line: pooled WMD between the two groups; *I*^2^, level of heterogeneity; *P* = 0.00 or less than 0.05, significant heterogeneity.

Subgroup analysis of continents showed no difference in IL-6 levels between patients with severe malaria and non-severe malaria among studies conducted in Africa (*P* = 0.121, WMD = 98.52 pg/mL, 95% CI =  − 26.02 –223.07 pg/mL, *I*^2^ = 100%, nine studies) and Asia (*P* = 0.073, WMD = 92.59 pg/mL, 95% CI =  − 8.69 –193.86 pg/mL, *I*^2^ = 99.7%, four studies, Fig. 3).

**Figure 4:**
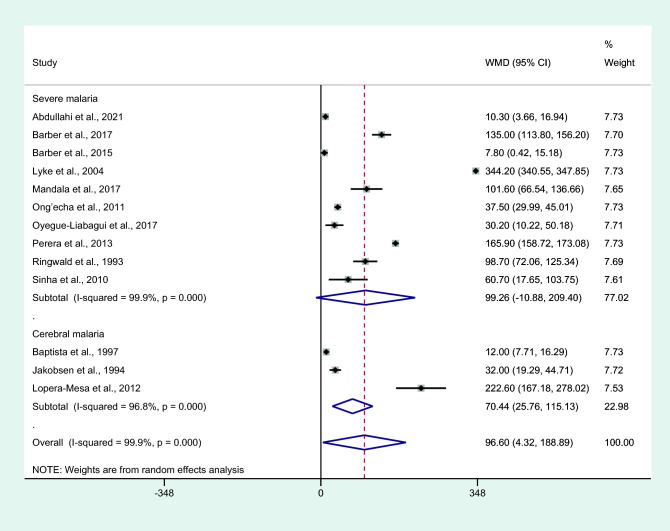
Differences in IL-6 levels between patients with severe malaria and non-severe malaria by complications. Abbreviation: WMD, weighted mean difference; CI, confidence interval; black diamond symbol, point estimate; solid line in the middle of the graph at 0, zero effect size; Dashed red line: pooled WMD between the two groups; *I*^2^, level of heterogeneity; *P* = 0.00 or less than 0.05, significant heterogeneity.

Subgroup analysis of malarial complications showed higher mean IL-6 levels in patients with cerebral malaria than in those with non-severe malaria (*P* = 0.002, WMD = 70.44 pg/mL, 95% CI = 25.76–115.13 pg/mL, *I*^2^ = 99.7%, three studies). No differences in IL-6 levels were observed between patients whose severe complications were not specified and those with non-severe malaria (*P* = 0.077, WMD = 99.26 pg/mL, 95% CI =  − 10.88 –209.4 pg/mL, *I*^2^ = 99.9%, 10 studies; Fig. 4).

**Figure 5:**
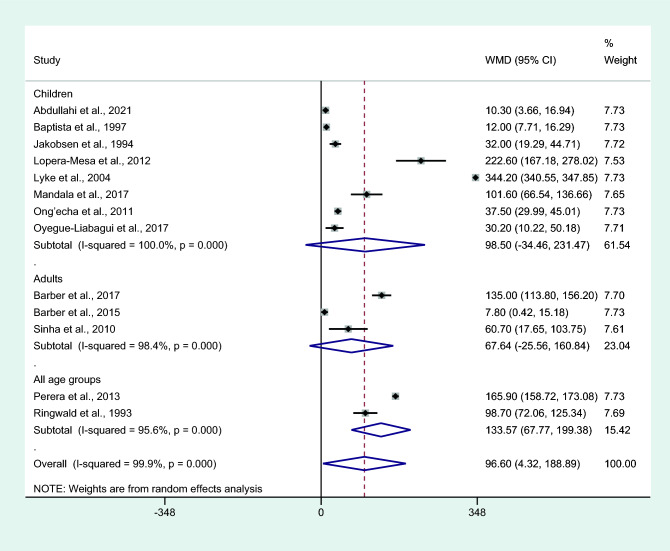
Differences in IL-6 levels between patients with severe malaria and non-severe malaria by age groups. Abbreviation: WMD, weighted mean difference; CI, confidence interval; black diamond symbol, point estimate; solid line in the middle of the graph at 0, zero effect size; Dashed red line: pooled WMD between the two groups; *I*^2^, level of heterogeneity; *P* = 0.00 or less than 0.05, significant heterogeneity.

Subgroup analysis of age groups showed higher mean IL-6 levels in those with severe malaria than in those with non-severe malaria among studies that enrolled patients of all age groups (*P* < 0.001, WMD = 133.57 pg/mL, 95% CI = 67.77−199.38 pg/mL, *I*^2^ = 95.6%, two studies). No differences in IL-6 levels between patients with severe malaria and non-severe malaria among the studies that enrolled only children (*P* = 0.147, WMD = 98.5 pg/mL, 95% CI =  − 34.46 −231.47 pg/mL, *I*^2^ = 100%, eight studies) and only adults (*P* = 0.155, WMD = 67.64 pg/mL, 95% CI =  − 25.56 −160.84 pg/mL, *I*^2^ = 98.4%, three studies; Fig. 5) were observed.

**Figure 6:**
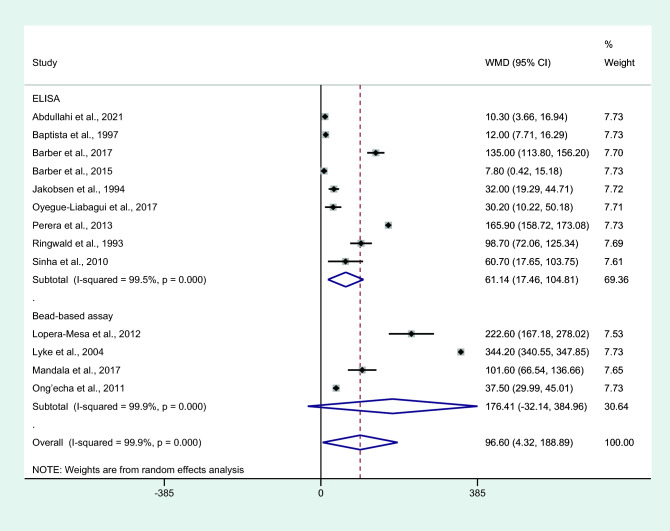
Differences in IL-6 levels between patients with severe malaria and non-severe malaria by assays for IL-6. Abbreviation: WMD, weighted mean difference; CI, confidence interval; black diamond symbol, point estimate; solid line in the middle of the graph at 0, zero effect size; Dashed red line: pooled WMD between the two groups; *I*^2^, level of heterogeneity; *P* = 0.000 or less than 0.05, significant heterogeneity.

Subgroup analysis of assays used to determine IL-6 levels showed a higher mean IL-6 level in cases with severe malaria than those with non-severe malaria among studies using an ELISA for IL-6 measurement (*P* = 0.006, WMD = 61.14 pg/mL, 95% CI = 17.46–104.81 pg/mL, *I*^2^ = 99.5%, nine studies). No difference in IL-6 levels between patients with severe malaria and non-severe malaria among studies using bead-based assays for IL-6 measurement was observed (*P* = 0.097, WMD = 176.41 pg/mL, 95% CI =  − 32.14 –38.96 pg/mL, *I*^2^ = 99.9%, four studies; Fig. 6).

### Differences in IL-6 levels between uncomplicated malaria and controls

**Figure 7:**
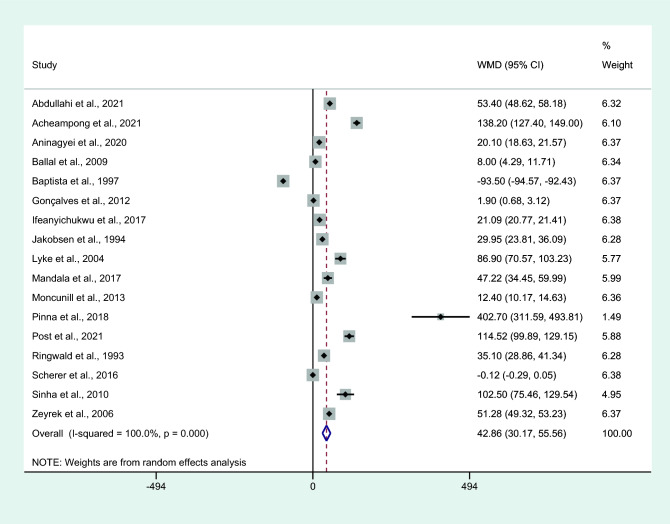
Differences in IL-6 levels between patients with uncomplicated malaria and controls. Abbreviation: WMD, weighted mean difference; CI, confidence interval; black diamond symbol, point estimate; solid line in the middle of the graph at 0, zero effect size; Dashed red line: pooled WMD between the two groups; *I*^2^, level of heterogeneity; *P* = 0.000 or less than 0.05, significant heterogeneity.

Differences in IL-6 levels between patients with uncomplicated malaria and controls were estimated using the available data from 17 studies^4,7,25–28,31,34,38,42,43,45,50,51,54,60,61^**.** Results showed that patients with uncomplicated malaria had higher mean IL-6 levels than controls (*P* < 0.001, WMD = 42.86 pg/mL, 95% CI = 30.17 − 55.56 pg/mL, *I*^2^ = 100%, 17 studies; Fig. 7). Meta-regression analyses of continents, *Plasmodium* species, parasitemia, age groups, male percentage, characteristics of the control groups, and IL-6 measurement methods showed that these co-variates did not confound the effect estimate (*P* > 0.05).

**Figure 8:**
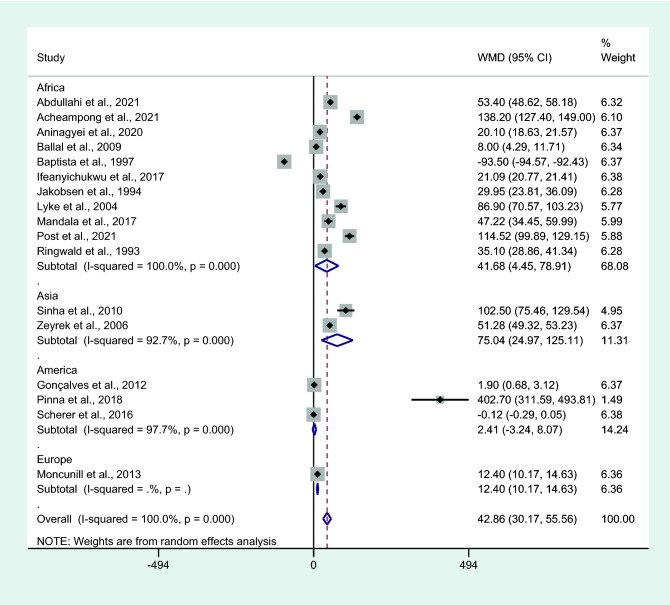
Differences in IL-6 levels between patients with uncomplicated malaria and controls by continents. Abbreviation: WMD, weighted mean difference; CI, confidence interval; black diamond symbol, point estimate; solid line in the middle of the graph at 0, zero effect size; Dashed red line: pooled WMD between the two groups; *I*^2^, level of heterogeneity; *P* = 0.000 or less than 0.05, significant heterogeneity.

Subgroup analysis of continents showed that the mean IL-6 levels were higher in patients with uncomplicated malaria than in controls among studies conducted in Africa (*P* = 0.028, WMD = 41.68 pg/mL, 95% CI = 4.45–78.91 pg/mL, *I*^2^ = 100%, 11 studies) and Asia (*P* = 0.003, WMD = 75.04 pg/mL, 95% CI = 24.97−125.11 pg/mL, *I*^2^ = 99.7%, two studies). No differences in IL-6 levels between patients with uncomplicated malaria and controls were observed among studies conducted in America (*P* = 0.403, WMD = 2.41 pg/mL, 95% CI =  − 3.24 –8.07 pg/mL, *I*^2^ = 97.9%, three studies; Fig. 8).

**Figure 9:**
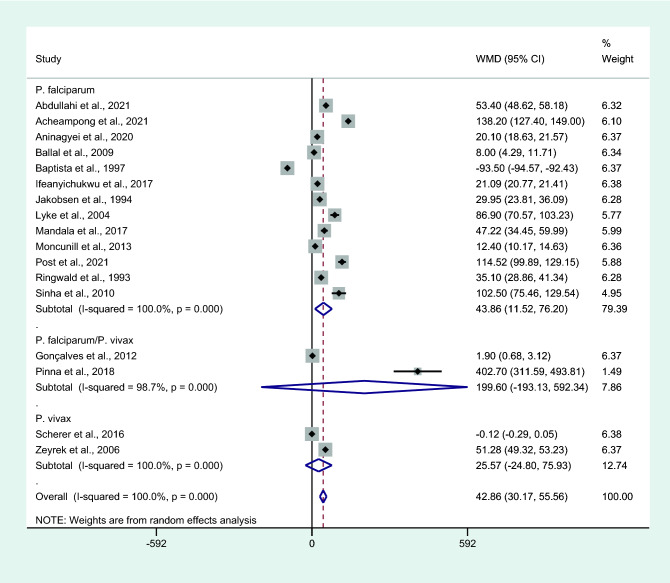
Differences in IL-6 levels between patients with uncomplicated malaria and controls by *Plasmodium* spp. Abbreviation: WMD, weighted mean difference; CI, confidence interval; black diamond symbol, point estimate; solid line in the middle of the graph at 0, zero effect size; Dashed red line: pooled WMD between the two groups; *I*^2^, level of heterogeneity; *P* = 0.000 or less than 0.05, significant heterogeneity.

Subgroup analysis of *Plasmodium* species showed higher mean IL-6 levels in patients with uncomplicated malaria than in controls among studies that enrolled patients infected with only *P. falciparum* (*P* = 0.008, WMD = 43.86 pg/mL, 95% CI = 11.52–76.2 pg/mL, *I*^2^ = 100%, 13 studies). No differences in IL-6 levels were observed between patients with uncomplicated malaria and controls among studies that enrolled patients infected with *P. falciparum/P. vivax* (*P* = 0.319, WMD = 199.6 pg/mL, 95% CI =  − 193.13 –592.3 pg/mL, *I*^2^ = 98.7%, two studies; Fig. 4) or only *P. vivax* (*P* = 0.32, WMD = 25.57 pg/mL, 95% CI =  − 24.8 −75.9 pg/mL, *I*^2^ = 100%, two studies; Fig. 9).

**Figure 10:**
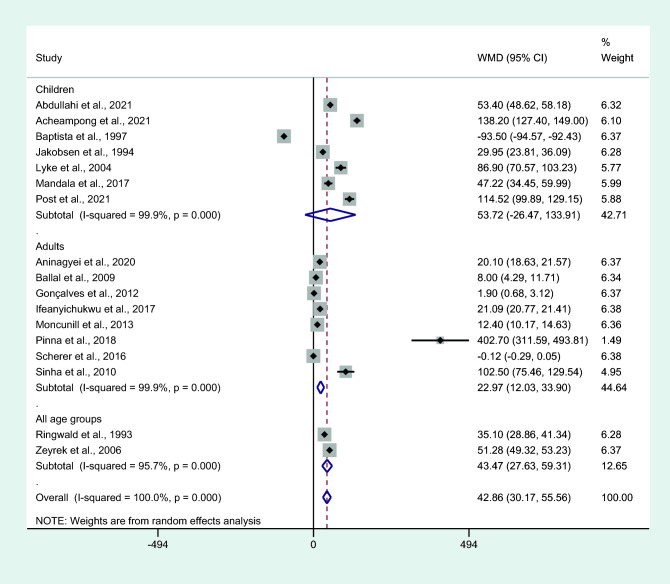
Differences in IL-6 levels between patients with uncomplicated malaria and controls by age groups. Abbreviation: WMD, weighted mean difference; CI, confidence interval; black diamond symbol, point estimate; solid line in the middle of the graph at 0, zero effect size; Dashed red line: pooled WMD between the two groups; *I*^2^, level of heterogeneity; *P* = 0.000 or less than 0.05, significant heterogeneity.

Subgroup analysis of age groups showed higher mean IL-6 levels in people with uncomplicated malaria than in controls among studies that enrolled patients of all age groups (*P* < 0.001, WMD = 43.47 pg/mL, 95% CI = 27.63 − 59.31 pg/mL, *I*^2^ = 95.7%, two studies) or adults only (*P* < 0.001, WMD = 22.97 pg/mL, 95% CI = 12.03–33.9 pg/mL, *I*^2^ = 99.9%, eight studies). No differences in IL-6 levels were observed between patients with uncomplicated malaria and controls among studies that enrolled only children (*P* = 0.189, WMD = 53.72 pg/mL, 95% CI =  − 26.47–133.91 pg/mL, *I*^2^ = 100%, seven studies, Fig. 10).

**Figure 11:**
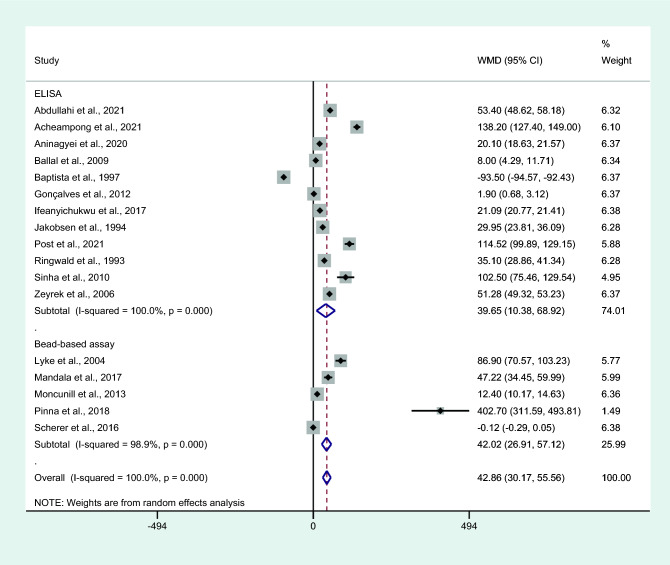
Differences in IL-6 levels between patients with uncomplicated malaria and controls by assays for IL-6. Abbreviation: WMD, weighted mean difference; CI, confidence interval; black diamond symbol, point estimate; solid line in the middle of the graph at 0, zero effect size; Dashed red line: pooled WMD between the two groups; *I*^2^, level of heterogeneity; *P* = 0.000 or less than 0.05, significant heterogeneity.

Subgroup analysis of assays used to measure IL-6 showed higher mean IL-6 levels in those with uncomplicated malaria than in controls among studies using an ELISA for IL-6 measurement (*P* = 0.008, WMD = 39.65 pg/mL, 95% CI = 10.38−68.92 pg/mL, *I*^2^ = 100%, 12 studies) and studies using bead-based assays for IL-6 measurement (*P* < 0.001, WMD = 42.02 pg/mL, 95% CI = 26.91–57.12 pg/mL, *I*^2^ = 98.9%, five studies; Fig. 11).

**Figure 12:**
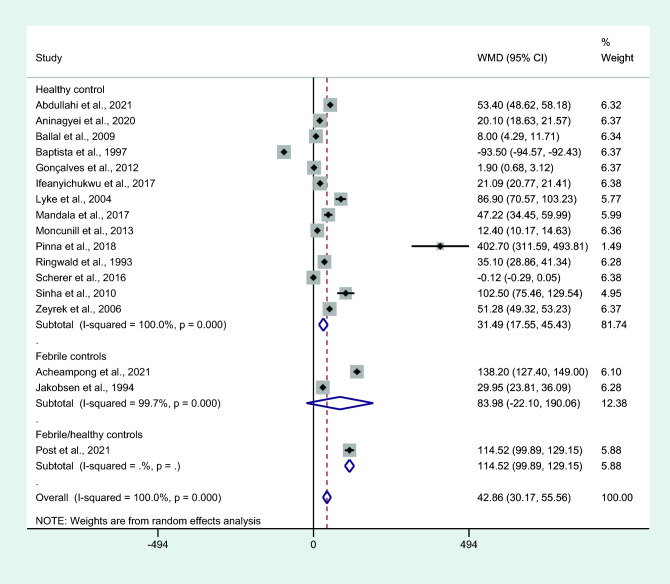
Differences in IL-6 levels between patients with uncomplicated malaria and controls by types of controls. Abbreviation: WMD, weighted mean difference; CI, confidence interval; black diamond symbol, point estimate; solid line in the middle of the graph at 0, zero effect size; Dashed red line: pooled WMD between the two groups; *I*^2^, level of heterogeneity; *P* = 0.000 or less than 0.05, significant heterogeneity.

Subgroup analysis of the characteristics of the control groups had higher mean IL-6 levels in those with uncomplicated malaria than in healthy controls (*P* < 0.001, WMD = 42.02 pg/mL, 95% CI = 26.91–57.12 pg/mL, *I*^2^ = 98.9%, 14 studies; Fig. 12). No differences in IL-6 levels were observed between patients with uncomplicated malaria and febrile controls (*P* = 0.121, WMD = 83.98 pg/mL, 95% CI =  − 22.1–90.1 pg/mL, *I*^2^ = 99.7%, two studies; Fig. 12).

### Differences in IL-6 levels between uncomplicated malaria and asymptomatic malaria

**Figure 13:**
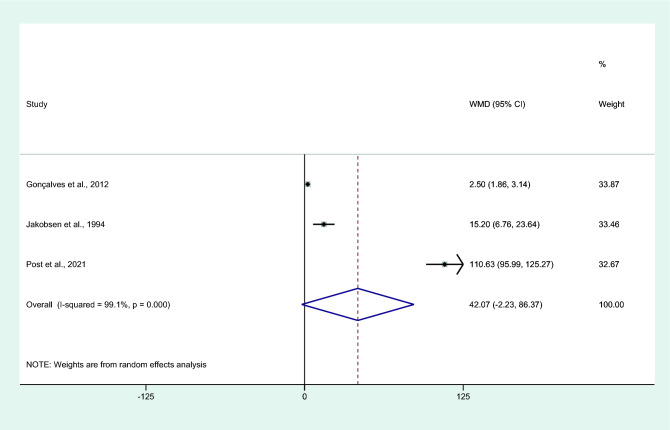
Differences in IL-6 levels between patients with uncomplicated malaria and asymptomatic malaria. Abbreviation: WMD, weighted mean difference; CI, confidence interval; black diamond symbol, point estimate; solid line in the middle of the graph at 0, zero effect size; Dashed red line: pooled WMD between the two groups; *I*^2^, level of heterogeneity; *P* = 0.000 or less than 0.05, significant heterogeneity.

Differences in IL-6 levels between patients with uncomplicated malaria and asymptomatic malaria were estimated using the available data of three studies^31,34,43^. Results showed no differences in the mean IL-6 levels between patients with uncomplicated malaria and those with asymptomatic malaria (*P* = 0.063, WMD = 42.07 pg/mL, 95% CI =  − 2.23 –86.37 pg/mL, *I*^2^ = 99.1%, eight studies; Fig. 13).

### Differences in IL-6 levels between asymptomatic malaria and healthy controls

**Figure 14:**
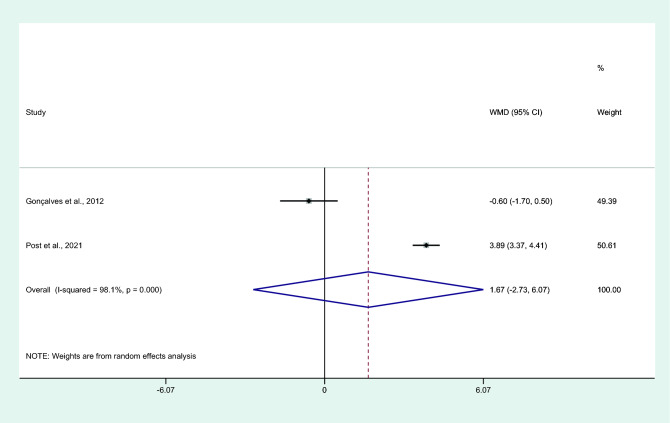
Differences in IL-6 levels between patients with asymptomatic malaria and controls. Abbreviation: WMD, weighted mean difference; CI, confidence interval; black diamond symbol, point estimate; solid line in the middle of the graph at 0, zero effect size; Dashed red line: pooled WMD between the two groups; *I*^2^, level of heterogeneity; *P* = 0.000 or less than 0.05, significant heterogeneity.

Differences in IL-6 levels between patients with asymptomatic malaria and healthy controls were estimated using the available data of two studies^31,43^. Results showed no differences in mean IL-6 levels between patients with asymptomatic malaria and healthy controls (*P* = 0.45, WMD = 1.67 pg/mL, 95% CI =  − 2.73 –6.07 pg/mL, *I*^2^ = 98.1%, two studies; Fig. 14).

### Differences in IL-6 levels between patients who died or survived

**Figure 15:**
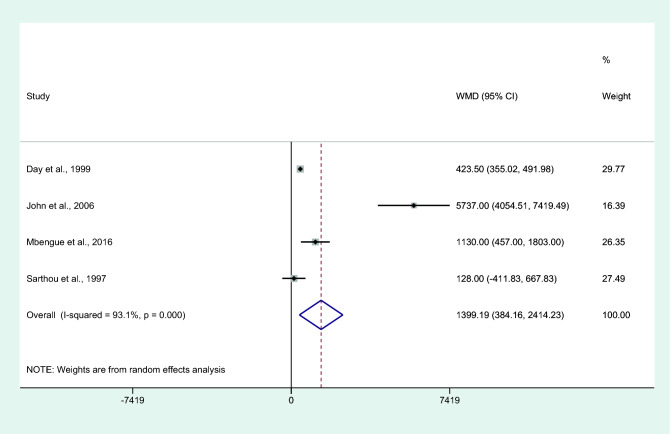
Differences in IL-6 levels between patients who died and those who survived. Abbreviation: WMD, weighted mean difference; CI, confidence interval; black diamond symbol, point estimate; solid line in the middle of the graph at 0, zero effect size; Dashed red line: pooled WMD between the two groups; *I*^2^, level of heterogeneity; *P* = 0.000 or less than 0.05, significant heterogeneity.

Differences in IL-6 levels between patients who died or survived were estimated using the available data of four studies^30,35,37,59^. Results showed higher mean IL-6 levels in patients who died than in those who survived (*P* = 0.007, WMD = 1399.19 pg/mL, 95% CI = 384.16– 2414.2 pg/mL, *I*^2^ = 93.1%, four studies. Figure 15). The summary of meta-analysis results is shown in Table 2.

**Table 2:** Summary of meta-analysis results.

Comparison	*P* value	WMD (pg/mL)	95% CI (pg/mL)	*I*^2^	Number of studies for estimation
Severe vs non-severe	0.04	96.63	0.88–19.38	99.9	13
Uncomplicated vs controls	< 0.001	42.86	30.17–55.56	100	17
Uncomplicated vs asymptomatic	0.063	42.07	− 2.23–86.37	99.1	8
Asymptomatic vs healthy controls	0.45	1.67	− 2.73–6.07	98.1	2
Died vs survived	0.007	1399.19	384.16–2414.2	93.1	4

*WMD* weighted mean difference, *CI* confidence interval.

### Publication bias

**Figure 16:**
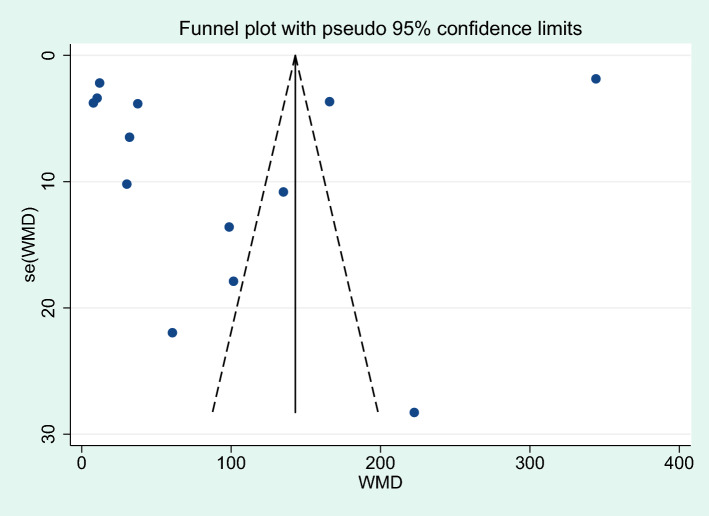
Funnel plot demonstrating the distribution of the effect size (WMD) and standard error of the effect size (seES) among studies included in the meta-analysis of severe and non-severe malaria.

**Figure 17:**
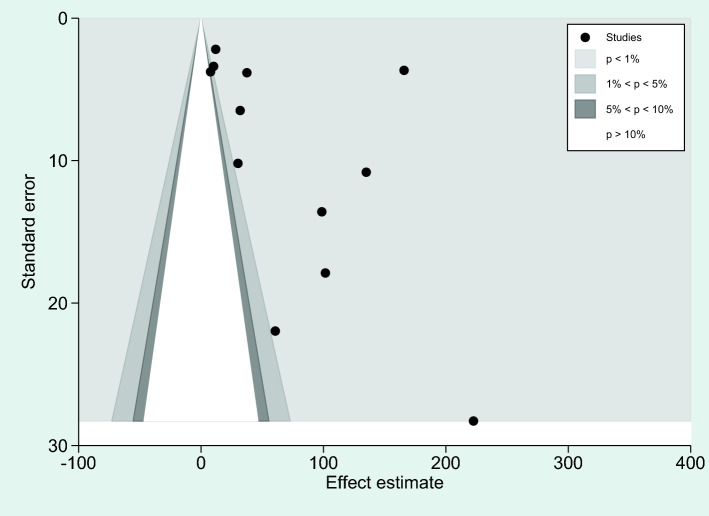
The contour-enhanced funnel plot demonstrated the effect estimates (WMD) were located in a significant area (*P* < 1%, 0.01), indicating that the cause of the funnel plot asymmetry was due to publication bias rather than other causes.

Publication bias was assessed with two analyses: differences in IL-6 levels between severe and non-severe malaria cases, and differences in IL-6 levels between uncomplicated malaria and controls. More than 10 studies were used in these analyses. For the analysis of differences in IL-6 levels between severe and non-severe malaria, a funnel plot demonstrated the asymmetrical distribution of effect estimates, which were far from the middle line of the plot (Fig. 16). Egger’s test demonstrated no significant small study effect (*P* = 0.365). A contour-enhanced funnel plot demonstrated that the effect estimates (WMD) were located in a significant area (*P* < 1%, 0.01, Fig. 17), indicating that the cause of the funnel plot asymmetry was due to publication bias rather than other causes. The trim and fill method was applied to correct the pooled effect estimate due to publication bias. The results showed that the pooled effect estimate was unchanged after the publication bias was adjusted (*P* = 0.04, WMD = 96.60 pg/mL, 95% CI = 4.32–188.89 pg/mL, 13 studies).

**Figure 18:**
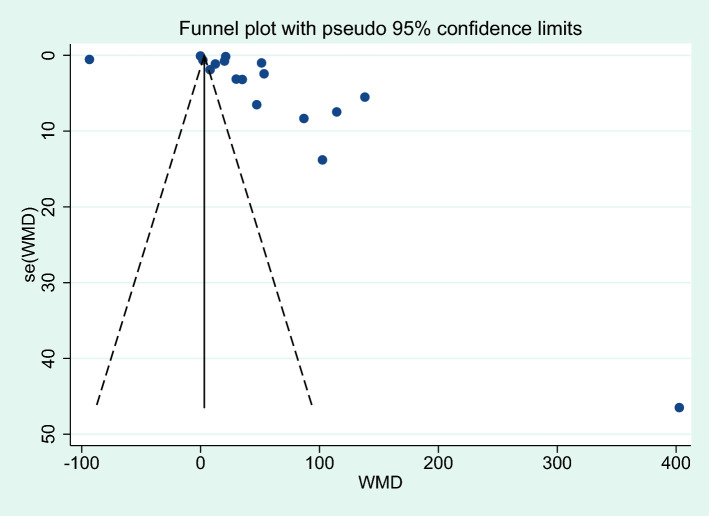
Funnel plot demonstrating the distribution of the effect size (WMD) and standard error of the effect size (seES) among studies included in the meta-analysis of uncomplicated malaria and controls.

**Figure 19:**
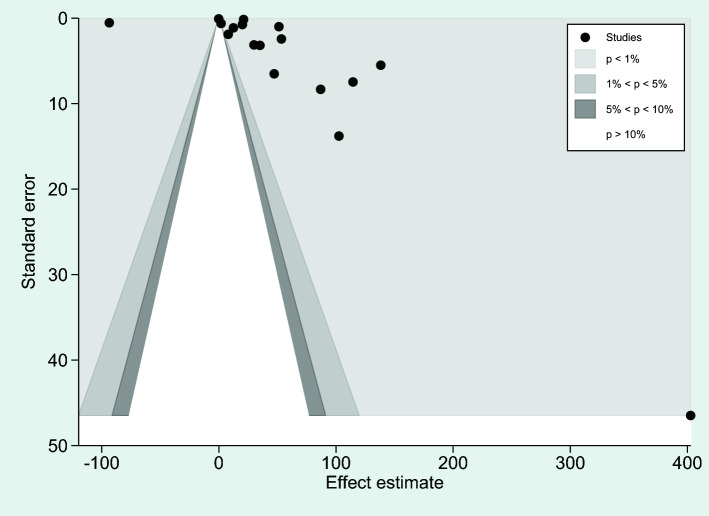
The contour-enhanced funnel plot demonstrated the effect estimates (WMD) were located in a significant area (*P* < 1%, 0.01), indicating that the cause of the funnel plot asymmetry was due to publication bias rather than other causes.

Regarding the analysis of differences in IL-6 levels between uncomplicated malaria and controls, a funnel plot showed an asymmetrical distribution of effect estimates far from the middle line (Fig. 18). Egger’s test demonstrated no significant small study effect (*P* = 0.686). The contour-enhanced funnel plot demonstrated that the effect estimates (WMD) were located in a significant area (*P* < 1%, 0.01, Fig. 19), indicating that the cause of the funnel plot asymmetry was due to publication bias rather than other causes. Results of the trim and fill method analysis showed that there was no difference in IL-6 levels between patients with uncomplicated malaria and controls after publication bias had been adjusted (*P* = 0.366, WMD = 5.122 pg/mL, 95% CI = -5.98 –16.23 pg/mL, 24 studies).

## Discussion

This systematic review aimed to determine whether IL-6 levels were associated with malaria infection and disease severity. The results based on the studies included in the meta-analysis indicated that IL-6 could be a marker for cerebral malaria as patients with severe malaria had higher mean IL-6 levels than those with non-severe malaria. Subgroup analysis of continents showed no differences in IL-6 levels between patients with severe malaria and non-severe malaria for studies conducted in Africa and Asia. In Africa, the highest difference in IL-6 levels was found in a study conducted in Mali^7^ and all studies included in the meta-analysis reported higher mean IL-6 levels in patients with malaria than patients with non-severe malaria^4,6,7,18,25,28,34,41,45^. Therefore, in Africa where malaria is endemic, high IL-6 levels are likely a useful marker for determining malaria severity. The study in Mali also demonstrated that elevated IL-6 levels were present in children that presented with cerebral malaria compared to those without cerebral malaria^7^. The result from that study was confirmed by a subgroup analysis that showed higher mean IL-6 levels in patients with cerebral malaria compared to those with non-severe malaria. In addition, the IL-6/parasite density ratio was highest in patients with clinical malaria, which suggested that high levels of IL-6 contributed to severe malaria, including cerebral malaria^62^. Although a previous study showed that malaria parasitemia was reported to be associated with elevated IL-6 levels^30^, our meta-regression analysis showed that the parasite count did not confound the WMD (pooled effect estimate), which supported the results of a previous study that indicated that the enrolled patients had a less acute form of severe malaria^7^.

Subgroup analysis of IL-6 measurement with an ELISA showed higher mean IL-6 levels in patients with severe malaria than in those with non-severe malaria. No differences in IL-6 levels were observed between patients with severe malaria and those with non-severe malaria when assessing studies that used bead-based assays for IL-6 measurements. The results of our meta-analysis demonstrated that using an ELISA for IL-6 measurements might be more useful than bead-based assays. Similar observations were demonstrated in a study measuring cytokine levels in the bronchoalveolar lavage of mice^63^, suggesting that an ELISA was more sensitive for detecting low levels of IL-6, whereas a bead assay was more suitable for measuring IL-6 levels in concentrated samples. Nevertheless, one study suggested that multiplex bead assays had better capability of detecting higher levels and, when using a multiplex bead assay, three times higher levels of cytokines were measured when compared with an ELISA^64^. In contrast to the results from comparisons between patients with severe malaria and those with non-severe malaria, the subgroup analysis of assays used for IL-6 measurements showed higher mean IL-6 levels in patients with uncomplicated malaria than in controls when an ELISA was used compared with bead-based assays. Nevertheless, without knowing the exact thresholds for each assay carried out in each laboratory for each type of ELISA/bead assay that the manufacturer used, further studies should use in-depth comparisons and clarify the differences in IL-6 levels between the two tests using the same blood samples.

Subgroup analysis of age groups showed higher mean IL-6 levels in those with severe malaria than in those with non-severe malaria among studies that enrolled patients of all age groups. However, no difference in IL-6 levels was found between patients with severe malaria and non-severe malaria in studies that enrolled only children or only adults. These results might be explained by the fact that only two studies^16,45^ were included in the subgroup of enrollment of patients in all age groups. The impact of age as an influential factor for IL-6 levels was demonstrated by a previous study that showed older children exhibited higher IL-6 levels and other Th1-biased chemokines levels when compared to younger children at the same stage of infection, which indicated that age is a dependent factor that contributes to inflammatory responses^65^. Nevertheless, the results of the meta-analysis suggested that higher mean IL-6 levels in patients with severe malaria compared to patients with non-severe malaria were independent of age, although a difference in IL-6 levels was reported between adults with severe malaria and those with non-severe malaria^17^. The meta-analysis results also showed higher mean IL-6 levels in patients who died than in those who survived. These significant differences were reported by three studies included in the meta-analysis^30,35,37^. The highest WMD was reported in a prospective observational study conducted in Uganda^35^. A potential explanation for these high IL-6 levels in patients might be because the study was conducted in children with cerebral malaria^35^, which supports a role for IL-6 in intense inflammation associated with cerebral malaria. Only one prospective observational study, conducted in Senegal, demonstrated no difference in IL-6 levels between two groups of patients. Therefore, further studies investigating the role of IL-6 in malaria-related mortality are needed.

Results of the meta-analysis showed that patients with uncomplicated malaria had higher mean IL-6 levels than controls. A subgroup analysis of continents showed that mean IL-6 levels were higher in patients with uncomplicated malaria than in controls among studies conducted in Africa and Asia. No differences in IL-6 levels, however, between patients with uncomplicated malaria and controls were observed in studies conducted in America. Furthermore, two studies in America^31,60^ reported no difference in mean IL-6 levels between two groups of patients and one study^42^ reported higher mean IL-6 levels in patients with uncomplicated malaria compared to healthy controls. A subgroup analysis of *Plasmodium* species showed higher mean IL-6 levels in patients with uncomplicated malaria than in controls among studies that enrolled patients infected with *P. falciparum* only. No differences in IL-6 levels between patients with uncomplicated malaria and controls were observed among studies that enrolled patients infected with *P. falciparum/P. vivax* and *P. vivax* only. One of the included studies reported that patients with *P. vivax* and *P. falciparum* infections had similar cytokine profiles; that is, increased levels of IL-6 and other cytokines, including IFN- γ, TNF-α, IL-8, IL-10, IL-13, MIP1b, and G-CSF. However, no differences in IL-6 levels were noted between two *Plasmodium* spp.^42^. A subgroup analysis of the characteristics of control groups showed higher mean IL-6 levels in those with uncomplicated malaria than in healthy controls, but no difference in IL-6 levels was observed between patients with uncomplicated malaria and febrile controls. These results might be due to studies that focused solely on microscopy failed to consider low density infected patients who had elevated IL-6 separately, further reducing the significance between uncomplicated malaria and febrile controls, as febrile control groups were not truly *Plasmodium* negative or with submicroscopic infection. Nevertheless, the meta-analysis results were in contrast to a case-controlled study that demonstrated patients with malarial fever had higher IL-6 levels than those with non-malaria fever^66^, indicating the increase in IL-6 levels might be specific for malaria compared to other febrile diseases. Previous studies showed that IL-6 concentrations in healthy individuals were typically low; that is, in the range of 0.2–7.8 pg/mL^67^. In one of the included studies^29^, IL-6 levels in healthy controls were below the detection limit of the ELISA method used, confirming that very low concentrations should be detected using a common method. Nevertheless, increased IL-6 levels in healthy individuals were reported to increase with age^68^. Moreover, IL-6 levels were reported to decrease with increasing transmission intensity, indicating that the transmission intensity was the predictor of IL-6 responses during acute malaria infections^69^. Based on the results of our meta-analysis and studies that were included in the analysis, IL-6 levels were increased in patients with uncomplicated malaria compared to healthy controls. This result indicated that IL-6 could be a candidate marker for diagnosing malaria infections. However, whether IL-6 levels are beneficial for differentiating malaria infections from other febrile diseases still needs to be elucidated by further studies.

Results of a meta-analysis showed no difference in mean IL-6 levels between patients with uncomplicated malaria and those with asymptomatic malaria. A study conducted in Burkina Faso^43^, however, showed a very high mean difference in IL-6 levels between two groups of participants. They clearly demonstrated that children with uncomplicated malaria had higher plasma cytokine levels, including IL-6, compared to those with asymptomatic malaria^43^. Furthermore, a previous study showed there was no difference in IL-6 levels between asymptomatic-infected and uninfected children^70^. Our met-analysis results showed no difference in IL-6 levels between patients with uncomplicated malaria and those with asymptomatic malaria and this might be due to the differences in the parasite density of patients with asymptomatic malaria as the mean parasitemia of patients with asymptomatic malaria was the lowest (mean 11.4 parasites per microliter) in a study conducted in Brazil^31^. The mean parasiemia of patients with asymptomatic malaria was higher in studies from Burkina Faso (mean 6,765 parasites per microliter)^43^ and in Gambia (mean 15,000 parasites per microliter)^34^. Also, the difference in methods for detecting malaria parasites might be attributed to inconsistency between the results of individual studies. A study from Brazil^31^ used both microscopy and PCR for detecting malaria parasites while another two studies^34,43^ used only microscopy. A previous study reported that IL-6 was positively associated with parasitemia and IL-6 was a predictor of parasitemia in children with microscopic asymptomatic malaria^71^. The null difference in mean IL-6 levels between patients with uncomplicated malaria and those with asymptomatic malaria might be due to *Plasmodium* spp. differences in individual studies. For example, the study in Brazil^31^ enrolled patients with *P. vivax* for the comparison of mean IL-6 levels between patients with uncomplicated malaria and those with asymptomatic malaria. Two other studies in Burkina Faso^43^ and Gambia^34^ enrolled patients with *P. falciparum* for comparisons of mean IL-6 levels between patients with uncomplicated malaria and those with asymptomatic malaria. A previous study also suggested that the higher parasitaemia thresholds for symptomatic malaria in malaria-endemic areas with intense malaria transmission might cause milder fevers or remain asymptomatic until higher parasite densities exist, and then the signs and symptoms were observed^69^. Taken together, the results of our meta-analysis showed that IL-6 levels were not different in patients with uncomplicated malaria and asymptomatic malaria. Therefore, it is recommended that future studies should investigate alterations of IL-6 levels in asymptomatic malaria to determine whether the IL-6 levels are altered in patients with asymptomatic malaria.

The present study has some limitations. First, there is heterogeneity of the estimate of the effect among the included studies, which affected the results of the meta-analyses. Second, there were a limited number of studies included in the meta-analysis that compared the differences in IL-6 levels between patients with uncomplicated and asymptomatic malaria, as well as asymptomatic malaria and healthy controls. Therefore, the meta-analysis results of these comparisons may have affected the robustness of the meta-analysis results.

## Conclusion

In conclusion, significantly increased levels of IL-6 were observed in patients with severe malaria compared with those in patients with non-severe malaria, indicating that IL-6 might be a candidate marker for severe malaria. In addition, IL-6 might be a candidate marker for malaria infection and therefore could be used to differentiate malaria infection from other febrile diseases. From the results of our study, we recommend that future studies should investigate the sensitivity and specificity of increased IL-6 levels to determine the effectiveness of measuring IL-6 levels to detect malaria infection and monitor malaria severity to improve the efficiency of malaria diagnosis and management.

## Supplementary Information

Supplementary Table S1.

Supplementary Table S2.

Supplementary Table S3.

## Data Availability

All data and related materials are presented in this manuscript.
