# Supplementary material for: Increased interleukin-6 levels associated with malaria infection and disease severity: a systematic review and meta-analysis
Source: Sci Rep. 2022 Apr 8;12:5982. doi: 10.1038/s41598-022-09848-9 (PMC8993930; doi:10.1038/s41598-022-09848-9)
Supplement: Supplementary file 1 — Supplementary Table S1. [file 41598_2022_9848_MOESM1_ESM.docx]

**Increased interleukin-6 levels associated with malaria infection and disease severity: A systematic review and meta-analysis**

Polrat Wilairatana^1^, Wanida Mala^2^, Giovanni De Jesus Milanez^3^, Frederick Ramirez Masangkay^4^, Kwuntida Uthaisar Kotepui^2^, Manas Kotepui^2*^

^1^Department of Clinical Tropical Medicine, Faculty of Tropical Medicine, Mahidol University, Bangkok, Thailand

^2^Medical Technology, School of Allied Health Sciences, Walailak University, Tha Sala, Nakhon Si Thammarat, Thailand

^3^ Department of Medical Technology, Faculty of Pharmacy, Royal and Pontifical University of Santo Tomas, Manila, Philippines

^4^Department of Medical Technology, Institute of Arts and Sciences, Far Eastern University – Manila, Manila, Philippines

**^*^Corresponding author**

Manas Kotepui; [manas.ko@wu.ac.th](mailto:manas.ko@wu.ac.th), Tel.: +66954392469

Polrat Wilairatana; [polrat.wil@mahidol.ac.th](mailto:polrat.wil@mahidol.ac.th)

Nuchpicha Intakhan; nuchpicha.in@wu.ac.th

Wanida Mala; [wanida.ma@wu.ac.th](mailto:wanida.ma@wu.ac.th)

Giovanni De Jesus Milanez; gmilanez81@gmail.com

Frederick Ramirez Masangkay; frederick_masangkay2002@yahoo.com

Kwuntida Uthaisar Kotepui; [kwuntida.ut@wu.ac.th](mailto:kwuntida.ut@wu.ac.th)

**Table S1. Search term**

| **Databases** | **Search terms/Search strategy** | **Date** |
| --- | --- | --- |
| MEDLINE (PubMed) | (malaria OR plasmodium) AND (IL-6 OR IL6 OR "Interleukin 6" OR "B-Cell Stimulatory Factor 2" OR "Differentiation Factor-2" OR BSF-2 OR "IFN-beta 2" OR "Interferon beta-2" OR MGI-2)  Search results: 506 | 7 November 2021 |
| Scopus | (malaria OR plasmodium) AND (IL-6 OR IL6 OR "Interleukin 6" OR "B-Cell Stimulatory Factor 2" OR "Differentiation Factor-2" OR BSF-2 OR "IFN-beta 2" OR "Interferon beta-2" OR MGI-2)  Search option: Title, abstract, keywords  Search results: 989 | 7 November 2021 |
| Web of Science | (malaria OR plasmodium) AND (IL-6 OR IL6 OR "Interleukin 6" OR "B-Cell Stimulatory Factor 2" OR "Differentiation Factor-2" OR BSF-2 OR "IFN-beta 2" OR "Interferon beta-2" OR MGI-2)  Search option: All fields  Search results: 474 | 7 November 2021 |
