# Supplementary material for: Increased interleukin-6 levels associated with malaria infection and disease severity: a systematic review and meta-analysis
Source: Sci Rep. 2022 Apr 8;12:5982. doi: 10.1038/s41598-022-09848-9 (PMC8993930; doi:10.1038/s41598-022-09848-9)
Supplement: Supplementary file 3 — Supplementary Table S3. [file 41598_2022_9848_MOESM3_ESM.docx]

**Increased interleukin-6 levels associated with malaria infection and disease severity: A systematic review and meta-analysis**

Polrat Wilairatana^1^, Wanida Mala^2^, Giovanni De Jesus Milanez^3^, Frederick Ramirez Masangkay^4^, Kwuntida Uthaisar Kotepui^2^, Manas Kotepui^2*^

^1^Department of Clinical Tropical Medicine, Faculty of Tropical Medicine, Mahidol University, Bangkok, Thailand

^2^Medical Technology, School of Allied Health Sciences, Walailak University, Tha Sala, Nakhon Si Thammarat, Thailand

^3^Department of Medical Technology, Faculty of Pharmacy, Royal and Pontifical University of Santo Tomas, Manila, Philippines

^4^Department of Medical Technology, Institute of Arts and Sciences, Far Eastern University – Manila, Manila, Philippines

**^*^Corresponding author**

Manas Kotepui: [manas.ko@wu.ac.th](mailto:manas.ko@wu.ac.th), Tel.: +66954392469

Polrat Wilairatana: [polrat.wil@mahidol.ac.th](mailto:polrat.wil@mahidol.ac.th)

Wanida Mala: [wanida.ma@wu.ac.th](mailto:wanida.ma@wu.ac.th)

Giovanni De Jesus Milanez: gmilanez81@gmail.com

Frederick Ramirez Masangkay: frederick_masangkay2002@yahoo.com

Kwuntida Uthaisar Kotepui: [kwuntida.ut@wu.ac.th](mailto:kwuntida.ut@wu.ac.th)

**Table S3. Quality the included studies**

|  | **Study** | **Score (out of 22)** | **Score (percentage)** | **Quality** |
| --- | --- | --- | --- | --- |
| 1. | Abdullahi et al., 2021  Case-control study | 19 | 86 | High |
| 2. | Acheampong et al., 2021  Case-control study | 17 | 77 | High |
| 3. | Aninagyei et al., 2020  Case-control study | 19 | 86 | High |
| 4. | Ballal et al., 2009  Case-control study | 15 | 68 | Moderate |
| 5. | baptista et al., 1997  Case-control study | 17 | 77 | High |
| 6. | Barber et al., 2015  Case–control study | 20 | 91 | High |
| 7. | Barber et al., 2017  Case–control study | 17 | 77 | High |
| 8. | Day et al., 1999  Case–control study | 17 | 77 | High |
| 9. | Dembele et al., 2016  Prospective observational study | 15 | 68 | Moderate |
| 10. | Goncalves et al., 2012  Prospective cohort study | 19 | 86 | High |
| 11. | Halsey et al., 2016  Retrospective study | 18 | 82 | High |
| 12. | Harawa et al., 2018  Prospective observational study | 18 | 82 | High |
| 13. | Hugosson et al., 2006  Prospective observational study | 16 | 73 | Moderate |
| 14. | Ifeanyichukwu et al., 2017  Cross-sectional study | 16 | 73 | Moderate |
| 15. | Jakobsen et al., 1994  Prospective observational study | 18 | 82 | High |
| 16. | John et al., 2006  Case–control study | 19 | 86 | High |
| 17. | Lopera-Mesa et al., 2012  Clinical trial/ Prospective cohort study | 21 | 95 | High |
| 18. | Lyke et al., 2004  Case–control study | 18 | 82 | High |
| 19. | Lyke et al., 2006  Prospective cohort study | 18 | 82 | High |
| 20. | MacMullin et al., 2012  Prospective observational study | 16 | 73 | Moderate |
| 21. | Mandala et al., 2017  Case–control study | 21 | 95 | High |
| 22. | Matiabe et al., 2020  Prospective observational study | 15 | 68 | Moderate |
| 23. | Mbengue et al., 2016  Prospective observational study | 19 | 86 | High |
| 24. | Moncunill et al., 2013  Prospective observational study | 19 | 86 | High |
| 25 | Mwanga-Amumpaire et al., 2015  Clinical trial | 22 | 100 | High |
| 26 | Nmorsi et al., 2010  Prospective observational study | 15 | 68 | Moderate |
| 27. | Olupot-Olupot et al., 2013  Prospective cohort study | 20 | 91 | High |
| 28. | Ong'echa et al., 2011  Prospective cohort study | 17 | 77 | High |
| 29. | Oyegue-Liabagui et al., 2017  Cross-sectional study | 18 | 82 | High |
| 30. | Perera et al., 2013  Prospective observational study | 19 | 86 | High |
| 31. | Pinna et al., 2018  Prospective observational study | 17 | 77 | High |
| 32. | Poluga et al., 2012  Prospective observational study | 16 | 73 | Moderate |
| 33. | Post et al., 2021  Cross-sectional study | 22 | 100 | High |
| 34. | Punnath et al., 2019  Case–control study | 20 | 91 | High |
| 35. | Ringwald et al., 1993  Prospective observational study | 18 | 82 | High |
| 36. | Saïssy et al., 1994  Prospective observational study | 17 | 77 | High |
| 37. | Sarthou et al., 1997  Prospective observational study | 15 | 68 | Moderate |
| 38. | Scherer et al., 2016  Cross-sectional study | 16 | 73 | Moderate |
| 39. | Sinha et al., 2010  Case–control study | 15 | 68 | Moderate |
| 40. | Thuma et al., 1996  Retrospective study | 19 | 86 | High |
| 41. | Wenisch et al., 1996  Prospective observational study | 17 | 77 | High |
| 42. | Yeom et al., 2003  Retrospective study | 19 | 86 | High |
| 43. | Zeyrek et al., 2006  Prospective observational study | 18 | 82 | High |

STROBE: Strengthening the Reporting of Observational Studies in Epidemiology
